# Initial co-ideation phase of a shared decision-making tool aimed at promoting physical activity in primary care: views expressed by patients, academics, and healthcare professionals

**DOI:** 10.3389/fpubh.2025.1483035

**Published:** 2025-05-30

**Authors:** Maria Valle Ramirez-Duran, Lola Prieto-López, Cristina Antón-Rodríguez, Andrés García-Ramos, Diana Monge-Martín, Juan Gómez-Salgado, Valle Coronado-Vázquez

**Affiliations:** ^1^Department of Nursing, University of Extremadura, Badajoz, Spain; ^2^Faculty of Medicine. Universidad Francisco de Vitoria, Pozuelo de Alarcón, Spain; ^3^Department of Sociology, Social Work and Public Health. Faculty of Labour Sciences, University of Huelva, Huelva, Spain; ^4^Safety and Health Posgraduate Program, Escuela de Posgrado, Universidad de Especialidades Espíritu Santo, Guayaquil, Ecuador; ^5^Las Cortes Health Centre, Madrid Health Service, Madrid, Spain

**Keywords:** shared decision-making, physical activity, health promotion, primary care, healthcare professionals, co-ideation

## Abstract

**Introduction:**

Although physical activity is a key determinant of health, it is not yet sufficiently promoted in primary care due to communication barriers and a lack of decision-making tools tailored to patients’ preferences.

**Objectives:**

The aim of this study was to explore the views, needs, and expectations of patients, academics, and healthcare professionals regarding physical activity and shared decision-making in primary care, as part of the initial phase of co-ideation for the development of a shared decision-making tool.

**Methods:**

This was a qualitative study based on focus groups. Participants were selected through purposive sampling to ensure representation of patients, academics, and healthcare professionals. Data were collected between June and December 2023 using a semi-structured interview guide. All focus groups were audio-recorded, transcribed verbatim, and analysed thematically using NVivo software.

**Results:**

Two topics emerged: (1) Perceptions of sedentary lifestyles and healthy physical activity, and (2) Views on a shared decision-making tool to promote healthy physical activity. Within the first topic, four categories emerged: Conceptions of sedentary lifestyle and healthy physical activity; Misconception of everyday activities as non-exercise; Exercise preferences and habits; and Barriers to exercise. Within the second topic, four other categories emerged: Functional value of SDM tools: personalisation, relevance, and shared responsibility; Collaborative dynamics between patients and professionals; Structural and relational barriers to shared decision-making such as time constraints in consultations and lack of training in motivational communication and Implementation difficulties related to digital literacy and access; Tailoring SDM tools: personalisation, inclusivity, and real-life applicability such simplifying the language, co-designing the tool with patients, and integrating it into routine clinical workflows were the recommended strategies to facilitate adoption.

**Conclusion:**

This initial co-ideation process revealed key user preferences and implementation challenges that should be considered when developing a shared decision-making tool for promoting physical activity in primary care. These findings highlight the importance of involving patients, professionals, and academics in the early stages of the design of the tool to improve its usability, acceptability, and impact on clinical practice.

## Introduction

Regular physical activity is essential in reducing the risk of cardiovascular disease, breast and colon cancer, osteoporosis and depression, as well as in managing diabetes, high blood pressure, dyslipidaemia, obesity, menopausal symptoms, and overall mortality (1). In addition, human health is interconnected with the health of the planet in that sustainable health initiatives often include the promotion of active lifestyles that reduce dependence on motorised transportation, thereby reducing carbon emissions and improving environmental health (2, 3). The global consensus agenda for 2030, which includes the Sustainable Development Goals (SDGs), recognises the importance of incorporating health into environmental sustainability. Promoting physical activity not only benefits individual health, but also contributes to broader environmental and public health goals (4, 5).

However, 27.5% of adults worldwide do not meet the WHO exercise guidelines (6). For adults and older adults, the WHO recommends 150–300 min per week of moderate aerobic activity or 75–150 min of vigorous aerobic activity weekly, plus muscle-strengthening exercises twice a week (7). The WHO Global Action Plan on Physical Activity 2018–2030 aims to reduce inactivity by 10% by 2025 and 15% by 2030, emphasising the importance of knowing the benefits of regular exercise (8). Health promotion and prevention activities are an integral part of interventions aimed at improving health and preventing disease, injury, and disability (9). These activities should be implemented across all settings and within the general scope of daily life (10–12). Consequently, diverse strategies to promote physical exercise have been explored, including direct advice—individual or in groups—, supervised or unsupervised physical activity, telephone or direct support, and motivational written information, albeit with mixed results (13, 14). Nonetheless, effective communication between healthcare professionals and patients is essential to engage patients in their own health care and to ensure adherence to treatments, thus fostering shared decision-making (15).

Shared decision-making (SDM) represents a collaborative approach between healthcare professionals and patients; it is applicable to any clinical act—whether diagnostic, therapeutic, or preventive—, and is particularly important when the benefits of an intervention are uncertain or the associated risks are substantial (16, 17). As part of ‘patient-centred care’, SDM prioritises a patient-centred care design that respects patients’ values and preferences, supported by a deliberative model that involves exchange of information and subsequent deliberation to determine the optimal choice (18). In clinical practice, SDM has been widely implemented in the management of chronic diseases such as diabetes, high blood pressure, and cancer, and has demonstrated positive results in improving patient understanding of treatment options and in encouraging greater engagement in healthcare (19, 20). Additionally, SDM is applied in acute disease scenarios and screening processes, where patients often express a desire to be informed and involved in health-related decisions (21–23). The essential components of SDM include the transmission of information (presenting options to the patient), attention to the patient’s values, and collaborative deliberation leading to a health-related decision (24). Elwyn et al. (25) operationalised SDM in clinical practice through a three-step model: ‘choice talk,’ where the healthcare professional makes sure that patients know that reasonable options are available; ‘option talk’, where more detailed information about these options is provided; and ‘decision talk’, which refers to supporting the work of considering preferences and deciding what is best.

Subsequently, a variety of SDM tools have been meticulously designed to detail the available alternatives while incorporating the latest evidence on their benefits and risks. In this study, the term ‘tool’ refers to any structured resource—digital or any other type—that supports SDM between patients and healthcare professionals. Current research in physical activity SDM tools is still limited, and the existing tools often fail to incorporate the views of all relevant stakeholders (26). These tools support SDM to achieve the goals of respecting personal values, improving knowledge of the disease, and reducing passivity in the selection of treatment options (27). In response to the growing development of decision support tools, the International Patient Decision Aid Standards (IPDAS) has established comprehensive guidelines including a checklist for professionals to assess the content, development process, and effectiveness of decision support tools based on established quality criteria (28). One of the criteria relates to the fact that the SDM tool has to be supported by current scientific evidence. In this study, the first phase of the development of the SDM tool included the review of clinical practice guidelines on healthy physical activity, identifying the key recommendations, the methodological quality of the guidelines, and their applicability.

Several studies have highlighted the application of decision support tools in the screening, diagnosis, and treatment of various conditions, demonstrating improvements in patient satisfaction through increased provision of information and greater participation in decision-making. However, their use in SDM remains limited. Several barriers to their use have been identified, including time constraints, insufficient knowledge, and inadequate clinical information systems that fail to incorporate these tools (29–31).

Addressing these gaps can lead to the creation of more effective, user-friendly tools that enhance patient engagement and adherence to healthy physical activity. Co-creation or collaborative processes involve the use of dialogue to generate and share ideas (co-ideation), design (co-design), implement (co-implementation), and evaluate new programmes (co-evaluation) (32). Co-ideation involves discussing the purpose of the project, including identifying barriers and collaborative solutions (33). In co-design, stakeholders and researchers collaborate in planning the research design. In co-implementation and co-evaluation, the involvement of stakeholders in data collection and evaluation helps to consolidate relationships and generate new innovative opportunities (32).

The purpose of co-creation in clinical research is the collaboration between researchers, service providers, and patients in order to bridge the gap between research and practice (34, 35). In this line, this study aimed to initiate a co-ideation process by conducting multiperspective qualitative research through focus groups with patients, healthcare professionals, and academics in order to explore shared values, needs, and expectations regarding physical activity and SDM in primary care.

In this phase, the focus was on the co-ideation of new knowledge related to stakeholders’ views and expectations, rather than on the co-design or development of a specific SDM tool.

## Methods

This study follows the guidelines set out in the Consolidated Criteria for Reporting Qualitative Research (COREQ) (36).

### Study design

A qualitative study was conducted using focus groups and an inductive thematic analysis approach. Qualitative methods allow for an in-depth exploration of people’s views, beliefs, and motivations. Giving voice to patients, healthcare professionals, and academics made it possible to capture their diverse needs and priorities, which is essential for fostering patient empowerment and informing the development of SDM tools. This approach aims to understand how individuals perceive and make use of SDM in everyday clinical contexts, using their own language and real-life examples (37).

### Research team

Four female researchers with experience in qualitative research and clinical work with chronically ill patients participated in this study. The research team included three physicians and one research nurse.

### Participants and sampling

Participants were selected by purposive sampling based on a theoretical sampling framework (38). For the inclusion of patients, a key criterion was their familiarity with healthcare professionals and their availability. Additionally, these patients were characterised by having one or more chronic conditions, making them frequent users of primary care services. This selection process was intended to cover a wide age range in order to capture a diverse set of opinions reflecting a variety of lifestyles. Moreover, all participants were required to have no physical disabilities that might impede their active participation. Primary care professionals (physicians and nurses) identified patients in routine consultations and invited those who met the inclusion criteria to participate. They were provided with verbal and written information about the purpose of the focus group and the topics to be discussed. Informed consent was requested before their inclusion in the groups.

Regarding the healthcare professionals, specialists in primary care were involved, contributing their experience in the health system and their knowledge of the day-to-day functioning of the system. The age group of professionals was intentionally narrowed by focusing on new or mid-career individuals, who were assumed to be particularly receptive and motivated to implement changes in their professional practice. The professionals were recruited from among the members of the health centre team.

Following the transcription of both patient and professional focus groups (FGs) discussions, a decision was made to incorporate academic experts in order to further saturate the data. This inclusion aimed to add a layer of interpretation concerning other areas potentially affected by exercise, such as pharmacotherapy and inflammatory conditions, where there exists a relative paucity of knowledge. They were fellow professors at the University of Extremadura.

### Data collection

Data were collected between June 2023 and December 2023. The sessions were conducted face-to-face in a meeting room at the Las Cortes Health Centre (Madrid) and at the University of Extremadura, always ensuring a quiet and private environment. Each session was audio-recorded with the prior consent of the participants. Field notes were also taken to capture non-verbal cues and group dynamics.

Four FGs—two including patients, one including healthcare professionals, and one including academics—were formed. Each group was moderated by an experienced qualitative researcher.

While participants did not interact directly across groups, their views were integrated thematically to foster a shared understanding of information in future collaborative stages.

Data were obtained using the same semi-structured topic guide in order to facilitate the discussion, which included the following topics: how to define sedentary lifestyle and healthy physical activity, healthy exercise habits and preferences, barriers to healthy physical activity, and participants’ experiences and expectations regarding SDM. The questions used in the guide were designed with the objective of the study in mind to capture key aspects of the participants’ experiences and perspectives.

Firstly, the moderator explained the function of an SDM tool to clarify how they work, as their usage is not currently widespread, and to illustrate how SDM can enable patient autonomy and promote individualised care. The intervention began by asking participants in the focus groups how they defined sedentary lifestyle and healthy physical activity, and then moved on to discussing their experiences, habits, and preferences regarding healthy physical activity and also their views on what is needed to lead an active life and the barriers to doing so. Subsequently, the questions focused on how participants understood SDM and how this approach could support patients and professionals in adhering to healthy physical activity. Finally, the advantages and obstacles of co-ideation for an SDM tool were discussed. The FGs with patients and healthcare professionals were held at the healthcare centre, and those involving academics, at the University. Also, open dialogue and exploratory questions were raised when necessary, and the moderator guided the discussion to ensure that no single individual dominated, thus allowing for a thorough exploration of the topics of interest. Before moving on to new topics, the moderator summarised the main conclusions, and participants were given the opportunity to express their agreement or suggest modifications. The moderator recorded field notes and memos throughout each focus group session, which lasted 1 h. At the conclusion of all FGs, the results were returned to all participants.

### Data analysis

All FG sessions were audio-recorded and the transcripts were meticulously reviewed to identify and correct any inaccuracies. The topic guide was used to develop a first draft of the initial code. Each question in the guide was associated with categories that served as the initial structure for the coding process. The data were first examined to identify patterns and emerging issues.

Topics emerging from the FG discussions were systematically organised using NVivo software by two coders. To ensure accuracy and interrater reliability, the researchers engaged in discussions to validate and confirm the identified topics and codes.

### Ethical considerations

This study protocol was approved by the Research Ethics Committee of the Fundación Jiménez Díaz on 03-14-2023, with code PIC070-23_OTROS.

All participants signed an informed consent form prior to participation and gave verbal consent to being recorded.

## Results

A total of 15 participants involved in four FGs were included in the study.

The details of the participants are listed in Table 1.

**Table 1:** Sociodemographic characteristics of participants (Spain, 2024).

Participant group	Age	Male	Female
Patients (n = 7)	Range: 52–82 years Mean: 69.1 years SD: 12.6	3	4
Academics (n = 3)	Range: 32–40 years Mean: 35.7 years SD: 4.0	-	3
Specialist in exercise and immune-physiology			1
Specialist in inflammatory chronic diseases			1
Specialist in in pharmacology			1
Professionals (n = 5)	Range: 25–48 years Mean: 30.3 years SD: 9.9	1	4
Specialists in primary healthcare		1	4

Two topics emerged:

1. Perceptions about sedentary lifestyle and healthy physical activity.
2. Views on an SDM tool to promote healthy physical activity.

Table 2 shows the different topics and matching sub-topics. Excerpts about the topic are detailed in Table 3.

**Table 2:** Topics and sub-topics (Spain, 2024).

Topics	Sub-topics
Perceptions about sedentary lifestyle and healthy physical activity.	Conceptions of sedentary lifestyle and healthy physical activity
	Misconception of everyday activities as non-exercise
	Exercise preferences and habits
	Struggling to stay active: emotional and practical barriers to physical activity
Views on the co-ideation of an SDM tool to promote healthy physical activity.	Functional value of SDM tools: personalisation, relevance, and shared responsibility
	Collaborative dynamics between patients and health professionals to promote physical activity
	Structural and relational barriers to implementing shared decision-making
	Tailoring SDM tools: ensuring personalisation, inclusivity and real-life applicability

**Table 3:** Excerpts from participants’ expressed perceptions about sedentary lifestyle and healthy physical activity (Spain, 2024).

Excerpt type	Group	Excerpt
Excerpts on sedentary lifestyle	Patients (p1–p7)	(p1) ‘Sitting still and inactive’.
		p3: ‘Walking for 30 min, at least that much. Doing at least the minimum you have been told to do’.
	Health professionals (p11–p15)	p14: ‘[…] It is a state of inactivity that is causing an increase in certain pathologies and an increase in the percentage of obesity in Spain’.
		(p15) ‘Sedentary lifestyle is an attitude of the society we have nowadays’.
	Academics (p8–p10)	p9: ‘Not moving’.
		p10: ‘[…] lack of physical activity’.
Excerpts on healthy physical activity	Patients (p1–p7)	p4: ‘The one that best aligns with your age, constitution, health characteristics, and personal preferences’.
		p3: ‘[…] a type of exercise defined as evolving from person to person’.
	Health Professionals (p11–p15)	p14: ‘[…] It very much depends on the individual, but it should be aerobic exercise plus strength training’.
		p15: ‘[…] It should be aerobic exercise plus strength training to achieve the best results’.
	Academics (p8–p10)	(p8) ‘It is recommended by WHO […] 3 days a week of moderate exercise which may include some of these intense exercises, depending also on the capabilities of the individual’.
		p10: ‘But it also depends very much on the intensity, the exercise, and the person’.
Excerpts on misconception	Patients (p1–p7)	(p2) ‘I suppose you could say this is a fairly sedentary lifestyle. It’s not just sitting on the couch watching TV, but regular activity has to go beyond that’.
		(p6) ‘I often think that I do enough moving around at home but I know that it is not enough exercise’.
	Health professionals (p11–p15)	(p11) ‘Integrating exercise into the daily routine is important, but it must be more structured to be effective’.
		(p14) ‘While walking to the supermarket is better than nothing, it does not meet the exercise criteria we promote for cardiovascular health’.
	Academics (p8–p10)	(p8) ‘Exercise should be structured and measured; it should not be just casual daily activity’.
		(p9) ‘Everyday activities rarely reach the necessary intensity to count as real exercise’.
Excerpts on preferences and habits	Patients (p1–p7)	p1: Well, walking and some specific suitable exercises for my personal problems, which is my back’.
		p2: ‘[…] I’m doing a bit more exercise now, but normally I just walk’.
		p4: ‘I go to the gym four times a week, about an hour each time. There’s one day I do cardio, on the cross trainer for fifty minutes or so, and there are three days I do more weight training’.
	Health professionals (p11–p15)	p12: ‘[…] I’m not consistent with exercise at all. […] weightlifting at home […] going for a walk a couple of days’.
		p15: ‘I am very irregular exercising. On a seasonal basis’.
	Academics (p8–p10)	p10: ‘I exercise 2 days a week or for an hour or an hour and a quarter. I do Spinning and then I climb a lot of stairs every day. 7 floors twice a day’.
		p8: ‘I do not do much exercise, to be honest […] I did play a lot of paddle tennis before, five days a week, I played paddle tennis, and it’s a two-hour match’.
		p10: ‘You have to train for exercise. […] You cannot start doing at 70 years of age what you have not done at 30. Now, if you have been doing sport all your life, at 70 you can do much more than a 30-year-old who has never done anything in their life. The body has to be trained to do sport because it adapts, logically it adapts, all organs, all systems, adapt to the demands of the exercise’.
Excerpts on barriers	Patients (p1–p7)	p4: ‘[…] tiredness. I have to get up really early […] and I arrive home in the afternoon exhausted. So the tiredness, the willpower to get out of it’.
		p2: ‘I stopped cycling last year because my knee started to hurt’.
		p5: ‘Motivation for me. To be stimulated. To have it as an obligation’.
	Health professionals (p11–p15)	p13: ‘Self-esteem […] it’s like a vicious circle, but… But a very difficult one for the patient’.
		p12: ‘[…] whatever pathology each person may have’.
	Academics (p8–p10)	p10: ‘I do not think we have the time, but really […] I could make time. What happens is that you have to have, well, a motivation that takes away the laziness to go and do it. Because I think that no matter how stressed you are, no matter how overwhelmed you are, you can find the time to do it, 45 min a day, you can find the time to do it’.
		p8: ‘One of the limitations is that swimming in summer is great, but in winter there are many places where there is no swimming pool’.

### Perceptions about sedentary lifestyle and healthy physical activity

#### Conceptions of sedentary lifestyle and healthy physical activity

Sedentary lifestyle was often described in terms of inadequate physical activity or daily movement that may not be vigorous or health-promoting.

Patients described it as the lack of engagement in any physical activity beyond ordinary daily tasks such as walking around the house or making short trips to nearby places. Some individuals emphasised the need to walk for at least walking 30 min a day to avoid a sedentary lifestyle.

Health professionals discussed sedentary lifestyle not only in terms of physical inactivity, but also considering the cultural and environmental factors that encourage or discourage active lifestyles and increase the risk of developing chronic diseases. Health professionals pointed out that modern conveniences and technologies contribute to a sedentary lifestyle by reducing the need for physical exertion in both work and leisure activities.

The academics agreed that sedentary lifestyle is fundamentally the lack of physical activity, mentioning that jobs requiring constant movement do not encourage a sedentary lifestyle.

In discussing healthy physical activity, patients offered a dynamic definition that emphasised the need for exercise that could be performed within the limitations of their health status and daily commitments. They concluded that as individuals improve their fitness, the intensity and duration of exercise should be adjusted accordingly.

Health professionals provided insights from a clinical perspective, in many cases directly pointing to health outcomes and prevention strategies. They discussed exercise as a prescription tailored to individual health needs, such as cardiovascular fitness or weight management. This perspective is based on clinical evidence and aims to maximise health benefits while minimizing risks.

Academics emphasised the adaptive nature of healthy physical activity and the need for it to be evidence-based. They discussed how definitions of what is considered healthy physical activity should evolve based on the latest research findings, which may influence the understanding of exercise intensity, duration, and frequency. They also highlighted the need for exercise recommendations to be culturally and contextually appropriate.

#### Misconception of everyday activities as non-exercise

During the discussion on sedentary lifestyles and healthy physical activity, a misconception emerged: the idea that simply being active in everyday or routine tasks is not considered beneficial exercise.

Patients discussed their daily activities and seemed to struggle with recognizing these activities as valid forms of exercise. A patient expressed the view that activities like walking to the store or performing household tasks should not count as exercise and did not acknowledge these actions as beneficial for their health in the way traditional exercise would be.

Health professionals reported on the importance of exercise and often mentioned the need for it to be more integrated into daily routines. However, even in this context, there seemed to be an underlying assumption that a more structured exercise routine is preferable. While they recognise the value of all types of physical movement, planned and more intense physical activity is often emphasised as the most beneficial.

Academics mostly defined exercise in a more structured way, focusing on the intensity and duration that qualify an activity as beneficial exercise. Their academic perspective may lean towards a more formal definition of exercise, which could inadvertently support the misconception among the general public that only certain types of physical activity can be considered meaningful exercise.

#### Exercise preferences and habits

Patients exhibited a range of exercise preferences and habits, ranging from low-impact, accessible exercise that can be easily integrated into daily routines without the need for specific equipment or environments to structured and high-intensity exercise performed in dedicated sports facilities. However, many patients identified walking and light exercise at home as their preferred activities, emphasising the importance of convenience and accessibility for it to be maintained in time.

Health professionals expressed a dual approach to their exercise habits and preferences. On the one hand, they recognised the value of incorporating physical activity into daily routines, similar to the views of patients. On the other hand, like the academics, they also emphasised the need for more structured exercise programmes that include moderate and high-impact activities.

Academics showed a preference for more structured and rigorous forms of exercise, often citing the importance of intensity in attaining effective health outcomes. They mentioned powerwalking, cycling and running, which require dedicated time and often special equipment or settings. This group exhibited a tendency to value the quantifiable benefits of exercise, reflecting a more formalised approach to fitness.

#### Barriers to exercise (struggling to stay active: emotional and practical barriers to physical activity)

The barriers are multifaceted and often intertwined, impacting patients, academics, and health professionals in distinct but overlapping ways.

Participants across all groups commonly cited time constraints as a significant barrier. Many expressed how the demands of work and family life leave little room for structured exercise. Patients mentioned that they often balance the management of their health condition with their daily responsibilities, and academics noted the difficulty in finding time to address their health needs around their professional commitments.

Physical limitations were another major barrier mentioned across the groups. Health professionals noted that chronic pain or injuries often prevent patients from engaging in standard forms of exercise. This was echoed by patients who shared their personal experiences of how physical discomfort or fear of aggravating an injury limited their activity options.

Motivation, or lack thereof, was a key barrier, particularly among patients and academics. Many participants acknowledged knowing the benefits of regular exercise but still found it difficult to get started or to maintain a routine. The lack of immediate, visible results from exercise was also mentioned as a demotivating factor.

Another barrier to participation in physical activity was the lack of access to suitable exercise environments, coupled with the economic barrier, where the cost of gym memberships or exercise equipment could be unaffordable for some individuals.

### Views on an SDM tool to promote healthy physical activity

#### Functional value of SDM tools: personalisation, relevance, and shared responsibility

Participants from all groups evaluated SDM tools in terms of facilitating the empowerment of individuals to take an active role in managing their health through physical exercise.

Patients appreciated the idea of a tool that could provide tailored recommendations and actively involve them in the planning process. They expressed that such involvement would make exercise plans more relevant and practical, increasing the likelihood of adherence. They also stressed the importance of feeling heard and that their preferences and limitations were considered, which they believed would increase their motivation.

Academics and health professionals also saw potential in SDM tools in improving the quality of exercise recommendations. They noted that these tools could integrate clinical data and patient-reported preferences to generate holistic plans that address individual health status, personal goals, and even psychological barriers. This could significantly improve the effectiveness of interventions by better adapting them to the patients’ actual needs and capabilities.

#### Collaborative dynamics between patients and health professionals to promote physical activity

Patients and health professionals agreed on the potential of these tools in improving patients’ adherence to exercise. Patients reported that they would feel more engaged in their own health management, as they would not only receive health advice, but would actively participate in the design of their exercise plans. Patients noted that, when involved in SDM, they would feel more responsible for the outcomes.

Health professionals also recognised the potential of these tools to streamline and enrich the consultation process if properly implemented. With access to detailed and personalised data from these tools, they can quickly assess a patient’s progress and tailor discussions more effectively. This could lead to more focused and productive interactions, where limited consultation time is used to address specific concerns and adjust exercise plans dynamically based on real-time data.

#### Structural and relational barriers to implementing shared decision-making

The main discussed barrier was time constrains. Many participants highlighted the limited time available during consultations, which poses a significant barrier to the implementation of individualised exercise recommendations through an SDM tool. For instance, health professionals mentioned that average consultations last only 5 min, making it challenging to provide detailed guidance on exercise.

Another recurring topic raised by health professionals was the difficulty in motivating patients to adhere to exercise programmes. Although the need for personalised exercise plans was recognised, and participants from all FGs agreed that generic advice is often ineffective, academics highlighted the complexity of tailoring exercise recommendations to individual health conditions and personal preferences.

Health professionals pointed out that the absence of appropriate implementation could result in these tools becoming a further source of complexity rather than facilitating an improvement in health outcomes. Thus, training for healthcare providers on how to use these tools effectively in their practice is essential. This way, it became evident that there is also a need for integrating these tools into existing healthcare workflows.

Patients mentioned that the relationship between patients and healthcare providers was deemed crucial for the success of any co-created tool, arguing that strong, long-term relationships with healthcare providers can enhance patient compliance and trust. Conversely, frequent changes of the healthcare providers who treat them at consultation can undermine patient trust and reduce the effectiveness of exercise interventions.

#### Tailoring SDM tools: ensuring personalisation, inclusivity and real-life applicability

Patients suggested that integrating the tool with personal schedules would help users identify convenient times for exercise. They also stressed the importance of customised exercise plans that consider individual preferences and limitations. They believed that such personalisation could make exercise less daunting and more feasible. P7 suggested incorporating social features to connect users with exercise partners, thus providing mutual encouragement and shared commitment.

From the academics’ perspective, they stressed the importance of the tool including evidence-based recommendations and, as healthcare professionals, a good patient-professional relationship to ensure credibility and effectiveness.

Furthermore, the discussions highlighted the importance of ensuring that these tools are inclusive and adaptable to diverse patient populations. This includes accounting for different age groups, cultural backgrounds, and levels of health literacy. Participants highlighted that for SDM tools to be effective, they must be accessible and user-friendly for all patients, providing customisable interfaces and content that can cater to a wide range of needs and preferences. Excerpts on the topic are detailed in Table 4.

**Table 4:** Excerpts from opinions on an SDM tool to promote healthy physical activity (Spain, 2024).

Excerpt type	Group	Excerpt
Excerpts on perceptions about SDM tools	Patients (p1–p7)	p4: ‘So it makes you co-responsible, it makes you feel that you are not an entity outside of your health where people give you pills, pills, pills’.
		p2: ‘They did all sorts of things to me, but nobody ever asked me why. Nobody asked me’.
	Health Professionals (p11–p15)	p13: ‘Support, motivation for them’.
		p11: ‘With a good decision-making process, maybe your doubts will be a little clearer’.
	Academics (p8–p10)	p10: ‘You can help by personalising’.
Excerpts on the impact of patient–health professional collaboration on promoting healthy physical activity during the co-ideation of the tool	Patients (p1–p7)	p4: ‘I think it helps to diagnose the possible needs of the patient in advance. Whether there is really a demand that the patient needs a tool to do healthy physical exercise, or they do not really need it’.
		p3: ‘[…] DNA of the project. For if you are going to present a tool for co-decision at the time of diagnosis, what does not make much sense is that the tool is not part of that co-decision. It would be very contradictory’.
	Health Professionals (p11–p15)	p14: ‘And feeling satisfied that you may have motivated your patient to do exercise, and maybe they did not do it before’.
Excerpts on challenges and constraints perceived during the co-ideation process	Patients (p1–p7)	p4: ‘It requires patient involvement’.
		p2: ‘[…] the actions taken must be very adapted’.
	Health Professionals (p11–p15)	p14: ‘To start with, the consultation lasts 5 min’.
		p15: ‘Patients are looking for quick solutions and solutions that do not require a lot of effort, and that everything is done and sorted out for them’.
	Academics (p8–p10)	p8: ‘You have to know what’s good and what’s bad for them, otherwise it might even be counterproductive. Studies have been done and there is still no agreement’.
		p10: ‘General scientific knowledge’.
Excerpts on recommendations to the co-ideation	Patients (p1–p7)	p7: ‘Having a reference group’.
		p2: ‘Adapted exercise’.
	Health Professionals (p11–p15)	p13: ‘Possibility to fill in preferences at home’.
		p12: ‘Making them aware of the problem’.
	Academics (p8–p10)	p8: ‘A good relationship with the professional’.
		p9: ‘Exercise can be monitored with wearable fitness watches’.

Across all focus groups, participants agreed on the importance of tailoring physical activity to individual capabilities and life contexts, and acknowledged the potential of SPM tools to further engage patients in physical activity through more personalised and motivating plans. While definitions and preferences varied slightly among stakeholders, there was broad consensus on key barriers, such as time constraints, limited motivation, and the need for a strong patient-professional relationship. Participants also shared common expectations about the tool: it should be adaptable, user-friendly, and grounded in both evidence and personal needs. These shared views point to the importance of co-designed solutions that are flexible, accessible, and meaningfully integrated into everyday clinical practice.

## Discussion

In this study, an initial co-ideation phase that explored the views of patients, health professionals and academics on the development of an SDM tool to promote healthy physical activity among chronically ill adult primary health care users was completed. The findings highlighted similar definitions about sedentary lifestyle and healthy physical activity and that adherence to an active life is challenging, but personalisation is a key component. A positive perception towards the co-ideation of the tool was found, noting some limitations along with recommendations to overcome them. This early co-ideation phase contributed to increasing stakeholder interest, which is associated with a positive impact on the use of the assessment results (39).

Participants defined sedentary lifestyle in terms of cultural and environmental factors that lead to an increased risk of developing of chronic diseases. The cultural context is crucial in understanding and addressing physical inactivity. Research suggests that beliefs and practices concerning physical activity are strongly influenced by cultural and social factors. Adapting physical activity guidelines to these cultural contexts can improve engagement and health outcomes, especially in minority and vulnerable communities (40, 41).

In discussing healthy physical activity, patients proposed a dynamic definition, emphasising exercises that are manageable within their health constraints and daily commitments. Thus, tailored exercise prescriptions are recommended to address individual health needs effectively (7, 42, 43). Additionally, higher levels of total physical activity have been associated with substantially reduced risk for premature mortality (44).

However, participants mentioned that adherence to an active lifestyle was difficult to achieve, with time constraints, lack of motivation or inadequate social support, among others, acting as barriers. These findings are also reported even when exercise programmes are prescribed by healthcare professionals (45). Moreover, long-term exercise adherence based on innovative approaches that promote physical activity remains a persistent concern.

Participants across all groups commonly cited time constraints as a key barrier. Physical limitations were another major obstacle, with health professionals noting that chronic pain or injuries often prevented patients from engaging in standard forms of exercise. Motivation, or rather the lack of it, was a critical barrier discussed, particularly by patients and academics. Additionally, the lack of access to suitable exercise environments and economic barriers, such as the cost of gym memberships or exercise equipment, were highlighted. Psychological barriers, particularly among health professionals, included negative self-perceptions about their bodies or abilities, which could deter participation in physical activities. These barriers are well-documented in the study by Spiteri et al. (46).

Regarding SDM, participants from all groups evaluated SDM tools as empowering individuals to take on an active role in managing their health through physical exercise. Since these views cannot be compared due to the lack of SDM tools for physical activity or exercise, those tools that are already in place are found to enable patients to engage in their health by increasing knowledge of their disease and improving overall satisfaction (47).

This study is subject to several limitations. Notably, it excludes functionally independent adults with mild cognitive impairments, thereby neglecting their perspectives. Furthermore, the research did not incorporate interviews with certain healthcare professionals, such as physiotherapists. Addressing these gaps in future research could enhance the robustness of the findings. To the authors’ knowledge, there is a lack of tools for SDM concerning physical exercise within primary healthcare settings, and limited research exploring professional and patient opinions on these tools, which contributes to the scarcity of relevant scientific literature. Furthermore, there is a lack of studies that explore the co-ideation of SDM tools (26).

The information gathered from patients, healthcare professionals, and academics constitutes a key step in the co-design of an SDM tool that is adapted to users’ needs to promote physical activity from primary care. The preferences, expectations, and barriers identified will contribute to both the structure and content of the tool, ensuring that it meets the real needs of users. For example, the participants’ emphasis on personalisation and contextual adaptation highlights the importance of designing interfaces and content that are flexible. The results also suggest that future research should explore strategies for integrating these tools into existing clinical workflows, including digital solutions and training modules for professionals. Specifically, the results of this initial co-ideation phase will guide the next steps of the co-creation process by defining the design requirements, content priorities, and implementation strategies to be addressed in subsequent collaborative workshops. These workshops will aim to translate stakeholder views into specific prototypes for testing, evaluation, and refinement.

In conclusion, the co-ideation of the project has provided insight into stakeholders’ experiences regarding physical activity and has highlighted the importance of research in SDM tools. This study emphasises the need for personalised, evidence-based recommendations on physical activity and the potential that SDM tools have for improving patient engagement and adherence to exercise. Addressing barriers to exercise and integrating these tools into routine healthcare practices may result in improved health outcomes in adults with chronic diseases.

## Data Availability

The raw data supporting the conclusions of this article will be made available by the authors, without undue reservation.
